# Epstein-Barr Virus Central Nervous System Infections and Mortality Risk in Patients Presenting With Suspected Meningitis: Results From the Botswana National Meningitis Survey and the Harare Meningitis Aetiology Study

**DOI:** 10.1093/ofid/ofaf660

**Published:** 2025-10-23

**Authors:** Jayne Ellis, James Milburn, Kebatshabile Ngoni, Christopher G Williams, Charles Muthoga, Taddy Mwarumba, Ezekiel Gwakuba, George Manenji, Rachita Suresh, Tichoaya Machiya, Janet Thubuka, Cassie Northcott, James Penney, Matthew Kinsella, Imogen Mechie, Samuel Ensor, Tshepo Leeme, Leah Owen, Hannah Barton, Keatlaretse Siamisang, Mark W Tenforde, William Hurt, Ronan Doyle, Daniel Grint, Síle F Molloy, Thomas S Harrison, David M Goldfarb, Madisa Mine, Margaret Mokomane, Gift Ngwende, Lenon Gwaunza, Tiny Mazhani, Chiratidzo Ndhlovu, Joseph N Jarvis

**Affiliations:** Clinical Research Department, London School of Hygiene and Tropical Medicine, London, United Kingdom; Clinical Research Department, London School of Hygiene and Tropical Medicine, London, United Kingdom; Botswana Harvard Health Partnership, Gaborone, Botswana; Botswana Harvard Health Partnership, Gaborone, Botswana; Botswana Harvard Health Partnership, Gaborone, Botswana; Botswana Harvard Health Partnership, Gaborone, Botswana; Department of Internal Medicine, University of Zimbabwe, Harare, Zimbabwe; Botswana Harvard Health Partnership, Gaborone, Botswana; Department of Internal Medicine, University of Zimbabwe, Harare, Zimbabwe; Botswana Harvard Health Partnership, Gaborone, Botswana; Princess Marina Hospital Laboratory, Gaborone, Botswana; Princess Marina Hospital Laboratory, Gaborone, Botswana; Botswana Harvard Health Partnership, Gaborone, Botswana; Botswana Harvard Health Partnership, Gaborone, Botswana; Botswana Harvard Health Partnership, Gaborone, Botswana; Botswana Harvard Health Partnership, Gaborone, Botswana; Botswana Harvard Health Partnership, Gaborone, Botswana; Botswana Harvard Health Partnership, Gaborone, Botswana; Botswana-UPenn Partnership, Gaborone, Botswana; Botswana-UPenn Partnership, Gaborone, Botswana; Botswana Harvard Health Partnership, Gaborone, Botswana; Department of Medicine, University of Washington School of Medicine, Seattle, Washington, USA; Botswana-UPenn Partnership, Gaborone, Botswana; Clinical Research Department, London School of Hygiene and Tropical Medicine, London, United Kingdom; Department of Infectious Disease Epidemiology and International Health, Faculty of Epidemiology and Population Health, London School of Hygiene and Tropical Medicine, London, United Kingdom; Institute for Infection and Immunity, City St George's University of London, London, United Kingdom; Institute for Infection and Immunity, City St George's University of London, London, United Kingdom; MRC Centre for Medical Mycology, University of Exeter, Exeter, United Kingdom; Department of Pathology and Laboratory Medicine, Faculty of Medicine, University of British Columbia, Vancouver, British Columbia, Canada; National Health Laboratory, Gaborone, Botswana; University of Botswana, Gaborone, Botswana; Department of Internal Medicine, University of Zimbabwe, Harare, Zimbabwe; Department of Internal Medicine, University of Zimbabwe, Harare, Zimbabwe; Department of Infectious Disease Epidemiology and International Health, Faculty of Epidemiology and Population Health, London School of Hygiene and Tropical Medicine, London, United Kingdom; Department of Internal Medicine, University of Zimbabwe, Harare, Zimbabwe; Clinical Research Department, London School of Hygiene and Tropical Medicine, London, United Kingdom; Botswana Harvard Health Partnership, Gaborone, Botswana

**Keywords:** cryptococcal, Epstein-Barr virus, HIV, meningitis, tuberculosis

## Abstract

**Background:**

Epstein-Barr virus (EBV) central nervous system (CNS) infection in immunocompromised hosts and among meningitis cohorts is well recognized. The clinical significance of EBV CNS infection, however, is poorly understood.

**Methods:**

Data were collected as part of the Botswana National Meningitis Survey and the Harare Meningitis Aetiology Study. The prevalence of EBV CNS infection (EBV DNA detected in cerebrospinal fluid [CSF] by means of quantitative polymerase chain reaction) was determined, alongside associations with baseline covariates and the in-hospital mortality rate.

**Results:**

A total of 581 participants with suspected meningitis were recruited. Of these, 54% were male, the median age (interquartile range) was 38 (29–46) years, and 76% were persons living with human immunodeficiency virus (HIV). Cryptococcal meningitis was the most common microbiologically confirmed infectious meningitis (12.0%), and 6.4% of participants had definite tuberculous meningitis. EBV CNS infection was common (26% [152 of 581]) and was associated with older age, being HIV positive, and CSF pleocytosis (*P* < .001). It was also associated with increased in-hospital mortality rate (odds ratio, 1.64, [95% confidence interval, 1.10–2.43]; *P* = .01), but after adjustment for sex, age, and HIV status, there was no longer evidence of an association (adjusted odds ratio, 1.29 [.84–1.98]; *P* = .25). In subgroup analyses, there was an indication that the association between EBV CNS infection and mortality rate may differ by meningitis subgroup.

**Conclusions:**

EBV CNS infection was common among our cohort, and it was strongly associated with CSF pleocytosis. Following multivariable analyses, EBV CNS infection overall was not associated with in-hospital mortality rate. EBV CNS infection in the context of meningitis is most likely a “bystander” virus that reflects heightened CSF inflammation.

## Teaser

Of 581 participants with suspected meningitis, 152 had Epstein-Barr virus (EBV) detected in cerebrospinal fluid (CSF). There was no association between EBV central nervous system infection and mortality rate, suggesting that EBV is a “bystander” virus reflecting heightened CSF inflammation.

Meningitis remains a major cause of disease and death among adults and children worldwide [1]. In sub-Saharan Africa, the majority of cases occur in people living with human immunodeficiency virus (HIV-1), in whom cryptococcal meningitis is the most frequent microbiologically confirmed cause, followed by tuberculous meningitis [2, 3]. The case fatality rate associated with these infections is devastatingly high (24%–50%), even in the context of clinical trials [4–7]. Undiagnosed coprevalent opportunistic infections—including central nervous system (CNS) human herpesviruses—may contribute to poor patient outcomes [8, 9].

The epidemiology of opportunistic CNS infections in sub-Saharan Africa, particularly viral infections, have historically been poorly characterized due to a lack of access to sensitive diagnostics [10]. While it is recognized that Epstein-Barr virus (EBV) CNS infection can cause a spectrum of CNS syndromes—including meningitis, encephalitis, and CNS lymphoma—neurological manifestations of EBV infections are reported to be rare, occurring in 0.5–7.5% of EBV infections globally [11]. However, in sub-Saharan Africa, where universal EBV seroconversion is often seen by age 3–4 years [12] and advanced HIV disease is commonly associated with human herpesvirus reactivations [10], EBV CNS infection may be more frequent. The few studies that have performed polymerase chain reaction (PCR) testing on cerebrospinal fluid (CSF) from patients presenting with symptoms of CNS infections have found high rates of EBV detection (13%–65%) [10, 13–17], particularly among adults living with HIV (36%–65%) [13–15, 17]. Among 708 adults with HIV-associated cryptococcal meningitis recruited as part of the AMBITION-cm randomized control trial, 27% (191 of 708) had detectable EBV in CSF [18].

The clinical implications and prognostic significance of EBV CNS infection in patients presenting with suspected meningitis is not well understood. EBV may cause neurological damage by direct invasion of brain cells [19] or trigger immune-mediated damage [20]; this could occur in the context of EBV CNS monoinfection, or EBV may amplify CNS damage [13] and impair clearance of pathogens in coinfection, leading to poorer outcomes [21]. In a retrospective cohort of Malawian adults living with HIV and diagnosed with confirmed bacterial meningitis, higher CSF EBV viral load (VL) was associated with an increased in-hospital mortality rate [13]. Conversely, PCR detection of EBV in the CSF may be a nonpathogenic marker of HIV-mediated immunosuppression, and EBV may present as a “bystander” virus following shedding from activated B lymphocytes trafficking across the blood-brain barrier and into the CSF during meningitis [10, 14].

The prevalence of EBV CNS monoinfection or coinfection among adults and children presenting with suspected meningitis has not, to our knowledge, been described elsewhere; and no studies to date have investigated whether EBV coinfection is associated with differential mortality rates across meningitis diagnostic groups. We report on the prevalence of EBV CNS infection among participants of the Botswana National Meningitis Survey (BNMS) and the Harare Meningitis Aetiology Study (HarMenAeS), and we present multivariable analyses of the association between EBV CNS infection, meningitis diagnostic group, and in-hospital mortality rate.

## METHODS

### Study Design

We analyzed clinical data and CSF samples collected prospectively from adults and children presenting with suspected meningitis to Princess Marina Hospital or Parirenyatwa Hospital recruited as part of the BNMS, or the HarMenAeS. The BNMS and HarMenAeS studies are 2 ongoing prospective cohort studies investigating meningitis etiology and temporal trends among adults and children presenting with suspected meningitis to 2 tertiary referral hospitals in Botswana and Zimbabwe. The studies have no exclusion criteria, and all patients who undergo diagnostic lumbar puncture to investigate for suspected meningitis are eligible for inclusion, irrespective of HIV status. The BNMS study recruited both children and adults, the HarMenAeS study only recruited adults =>18 years. Patients presenting between November 2016 and October 2023 were included in this study.

### Participants and Study Procedures

Descriptions of the cohorts have been presented elsewhere [22, 23]; children and adults (aged ≥18 years) presenting with suspected meningitis to Princess Marina Hospital, and adults (aged ≥18 years) presenting to Parirenyatwa Hospital underwent comprehensive CSF analyses to ascertain the causes of meningitis. All participants underwent CSF testing for microscopy and culture, cryptococcal antigen testing, and PCR for bacteria, viruses and *Cryptococcus neoformans*/*gattii* using the BioFire FilmArray Meningitis/Encephalitis panel (bioMérieux). The full panel of diagnostic tests, including CSF MTB/RIF Xpert Ultra (Cepheid) was dependent on receipt of a sufficient volume of CSF; therefore, not all tests were performed in all participants. Blood cultures and additional tuberculosis diagnostics were performed at the physician’s discretion.

Clinical and laboratory data and treatment outcomes were captured in real time. Patients received standard of care anti-infective therapy and antiretroviral therapy (ART) per Botswana and Zimbabwe National guidelines [24]. We collected baseline demographic and clinical data, including ART status, and laboratory data, including CSF results of microscopy, white blood cell (WBC) count, CSF protein and glucose levels, and CSF fungal and bacterial culture. Participants were followed up until hospital discharge.

### EBV Testing and Definitions

Only BNMS and HarMenAeS participants with stored CSF samples (collected during acute hospitalization) available for testing were included in this analysis. DNA was extracted from 500 µL of CSF using the AllPrep DNA/RNA Mini Kit (QIAGEN) kits, according to the manufacturer’s instructions. Single-plex EBV quantitative PCR was performed using the EBV R-GENE assay (bioMérieux) and the Applied Biosystems Quantstudio 6 flex System (Life Technologies). Laboratory staff processed coded samples and were blinded to participant data and outcomes.

EBV CNS infection was defined as anyone with detectable EBV DNA in CSF using the qualitative PCR detection threshold defined by the test manufacturer. The reported limit of detection of the EBV R-GENE assay is 182 copies/mL. EBV CNS coinfection was defined as the detection of EBV plus microbiological diagnosis of another meningitis pathogen.

To maximize precision for modeling purposes, 5 composite meningitis diagnostic groups were formed using well-established case definitions: (1) cryptococcal meningitis, defined as positive CSF cryptococcal antigen result, India ink stain and/or *Cryptococcus* CSF culture, or PCR for *C neoformans/gattii*; (2) tuberculous meningitis, further stratified into definite, probable or possible tuberculous meningitis using the Marais Criteria [25]; (3) bacterial meningitis, defined as positive bacterial CSF gram stain, culture, or PCR result, a bacterial pathogen in the blood with CSF pleocytosis (CSF WBC count ≥5/μL), or neutrophilic meningitis (CSF pleocytosis with >50% neutrophils); (4) viral meningitis, defined as viral pathogen identified with PCR or lymphocytic meningitis (CSF pleocytosis with >50% lymphocytes); and (5) other suspected meningitis, a category that included participants with suspected meningitis with no microbiological diagnosis and a CSF WBC count <5/μL. An inflammatory CSF profile was defined as a CSF WBC count ≥5/μL (CSF pleocytosis), a CSF protein level ≥4.5 mg/dL, and/or a CSF glucose level <2.5 mmol/L.

### Ethics Statement

Institutional review board approval for BNMS was provided by the Botswana Health Research Development Council (Botswana Ministry of Health and Wellness) and the University of Botswana, with local approval from Princess Marina Hospital. Approvals for HarMenAeS have been obtained from the Research Council of Zimbabwe, the Medical Research Council of Zimbabwe and the Joint Research and Ethics Committee, Parirenyatwa Group of Hospitals. Both studies were also approved by the London School of Hygiene and Tropical Medicine Institutional Review Board. BNMS and HarMenAeS are surveillance studies using routinely collected data; therefore, a waiver of individual patient consent was obtained for each study.

### Statistical Analyses

Baseline demographics and clinical characteristics were summarized as counts and percentages for categorical data or medians (with interquartile ranges [IQRs]) and means (with ranges) for continuous data, depending on data distributions. The proportions of participants with EBV monoinfection and coinfection were determined, and baseline variables (eg, sex, age, and HIV status) were compared by EBV infection coinfection status using χ^2^ tests; Mann-Whitney *U* or Student *t* tests were used for continuous data.

The primary outcome was in-hospital mortality. Logistic regression models were used to investigate the association between EBV infection status and this outcome. All baseline covariates were considered as potential confounders. Age, sex, and HIV status were included a priori; thereafter, only covariates associated with both EBV infection status and in-hospital mortality were included in the regression models. CSF WBC count, CSF protein and glucose concentrations, and the meningitis diagnostic group were considered to potentially be on the causal pathway and were therefore not subjected to adjustment (Supplementary Figure 1). A likelihood ratio test was conducted to assess for effect modification between EBV infection and the meningitis diagnostic subgroup.

An exploratory analysis was conducted to investigate whether there was an association between CSF EBV burden and in-hospital death. In clinical practice, there is currently no established threshold to distinguish high-level from low-level EBV infection. For the purposes of exploratory dose–response analysis, we therefore stratified participants into 3 groups based on the CSF EBV VL: negative, low, and high, using the median value of ≥1885 copies/mL as the threshold. Univariable and multivariable logistic regression models—with the same covariate structure described above—were used to assess potential dose–response associations across these categories. Recognizing the limitations of dichotomizing EBV VL by using a median cutoff, we also modeled EBV VL as a continuous variable to capture its full distribution and potential linear association with the outcome.

In the presence of missing data, only complete cases were used for multivariable analysis. We assessed the normality of continuous variables using visual inspection of histograms. Model fit for regression analyses was evaluated using the Hosmer-Lemeshow test. *P* values have not been adjusted for multiple comparisons. All analyses were performed using Stata software, version 16 (StataCorp).

## RESULTS

### Cohort Characteristics

Between 11 November 2016 and 22 October 2023, a total of 581 patients with suspected meningitis were recruited. Just over half (54% [309 of 571]) were male, and the median age (IQR) was 38 (29–46) years. More than three-quarters of the cohort (76% [387 of 506]) were living with HIV, including 80.7% of adults (377 of 467) and 15.1% of children (5 of 33). Among participants living with HIV, the median CD4 cell count was 183 cells/μL (IQR, 50–385 cells/μL), and 42% (161 of 387) were taking ART at the time of presentation. Patients presented with a range of clinical symptoms consistent with meningitis: 66% (288 of 437) reported headache, 54% (265 of 488) reported fever, 58% (276 of 472) had altered mental status, 36% (146 of 405) had neck stiffness, and 23% (93 of 405) reported seizures; 60% (237 of 394) had a Glasgow Coma Scale score <15 at baseline (Table 1).

**Table 1.** Baseline Characteristics of 581 Patients With Suspected Meningitis, Stratified by Epstein-Barr Virus Central Nervous System Coinfection Status

Variable at Baseline	No. Assessed^a^	Patients, No. (%)^b^			*P* Value^c^
		Total Cohort	EBV CNS Infection	No EBV CNS Infection	
Total	581	581	152 (26.2)	429 (73.8)	…
Male sex	571	309 (54.1)	82 (55.0)	227 (53.8)	.79
Adult (age ≥18 y)	565	498 (88.1)	143 (96.6)	355 (85.1)	<.001
Child (age <18 y)	565	67 (11.9)	5 (3.4)	62 (14.9)	<.001
Age, median age, y	565	38 (29–46)	39 (32–47)	37 (28–46)	.05
Headache	437	288 (65.9)	96 (76.2)	192 (61.7)	.004
Fever	488	265 (54.3)	60 (46.1)	205 (57.3)	.03
Altered mental status	472	276 (58.5)	75 (58.6)	201 (58.4)	.9
Seizures	405	93 (23.0)	19 (16.0)	74 (25.9)	.03
HIV parameters					
Living with HIV	506	387 (76.5)	118 (87.4)	269 (72.5)	<.001
On ART at diagnosis	387	161 (41.6)	47 (39.8)	114 (42.4)	.06
CD4 cell count, median (IQR), cells/μL	223	183 (50–385)	134 (50–371)	188 (50–388)	.78
CSF parameters					
Pleocytosis (WBC count >5/μL)	543	241 (41.5)	80 (52.6)	161 (37.5)	.001
Protein, median (IQR), mg/dL	342	.65 (0.35–1.4)	0.8 (0.45–2.0)	0.59 (0.31–1.3)	.002
Glucose, median (IQR), mmol/L	448	3.0 (2.1–3.7)	2.6 (1.1–3.4)	3.1 (2.4–3.8)	<.001
Diagnostic group					
Cryptococcal meningitis	581	70 (12.0)	27 (17.8)	43 (10.0)	<.001
Bacterial meningitis		48 (8.3)	19 (12.5)	29 (6.8)	
Tuberculous meningitis		76 (13.1)	26 (17.1)	50 (11.7)	
Viral meningitis		143 (24.6)	39 (25.7)	104 (24.2)	
Other suspected meningitis		244 (42.0)	41 (27.0)	203 (47.3)	

Abbreviations: ART, antiretroviral therapy; CNS, central nervous system; CSF, cerebrospinal fluid; HIV, human immunodeficiency virus; IQR, interquartile range; WBC, white blood cell.

^a^Data were missing for the following categories: sex, missing in 10 participants (1.7%); age, missing in 16 (2.7%); headache at baseline, missing in 144 (24.8%); fever at baseline, missing in 93 (16%); altered mental status at baseline, missing in 109 (18.8%); seizures at baseline, missing in 176 (30.2%); HIV status, missing in 75 (12.9%); CSF WBC count, missing in 38 (6.5%); CSF protein level, missing in 239 (41.1%); and CSF glucose level, missing in 133 (22.9%).

^b^Data represent no. (%) of participants unless otherwise noted.

^c^ *P* values based on χ^2^ tests for categorical data and Mann-Whitney *U* or Student *t* tests for continuous data.

Cryptococcal meningitis was the most common microbiologically confirmed etiology in 12.0% (70 of 581) of participants; 6.4% (37 of 581) had definite and 6.7% (39 of 581) had probable/possible tuberculous meningitis, 24.6% (143 of 581) had definite/probable viral meningitis, 8.3% (48 of 581) had definite/probable bacterial meningitis, and 42.0% (244 of 581) were categorized as other suspected meningitis (Table 2).

**Table 2.** Primary Infective Diagnoses in 581 Adults and Children Presenting With Suspected Meningitis

Diagnosis	Patients, No. (%) (%)		
	Total Cohort (n = 581)	Adults (n = 498)	Children (n = 67)
Cryptococcal meningitis^a^	70 (12.0)	65 (13.0)	1 (1.5)
Bacterial meningitis^b^	48 (8.3)	42 (8.4)	6 (8.9)
Tuberculous meningitis^c^	76 (13.1)	72 (14.4)	2 (2.9)
Viral meningitis^d^	143 (24.6)	115 (23.1)	24 (35.8)
Other suspected meningitis	244 (42.0)	204 (40.9)	34 (50.7)

^a^Five patients with cryptococcal meningitis had coinfection (with cytomegalovirus [CMV] in 3 and varicella zoster virus [VZV] in 2).

^b^Including 42 patients with a confirmed microbiological diagnosis of bacterial meningitis (of whom 4 had CMV coinfection) and 6 with neutrophilic pleocytosis of unknown cause.

^c^Including 37 patients with definite (1 with CMV coinfection with *Streptococcus pneumoniae* and 1 with human herpesvirus 6 [HHV-6] coinfection), 1 with probable, and 38 with possible tuberculous meningitis.

^d^Including 40 patients with viral meningitis confirmed using the BioFire FilmArray Meningitis/Encephalitis panel (bioMérieux; herpes simplex virus 1 and 2 in 1 each, VZV in 3, CMV in 10, human HHV-6 in 9, *Enterovirus* in 4, and VZV and HHV-6 in 1) and 103 with lymphocytic pleocytosis of unknown cause.

### EBV Coinfections

**Figure 1.**
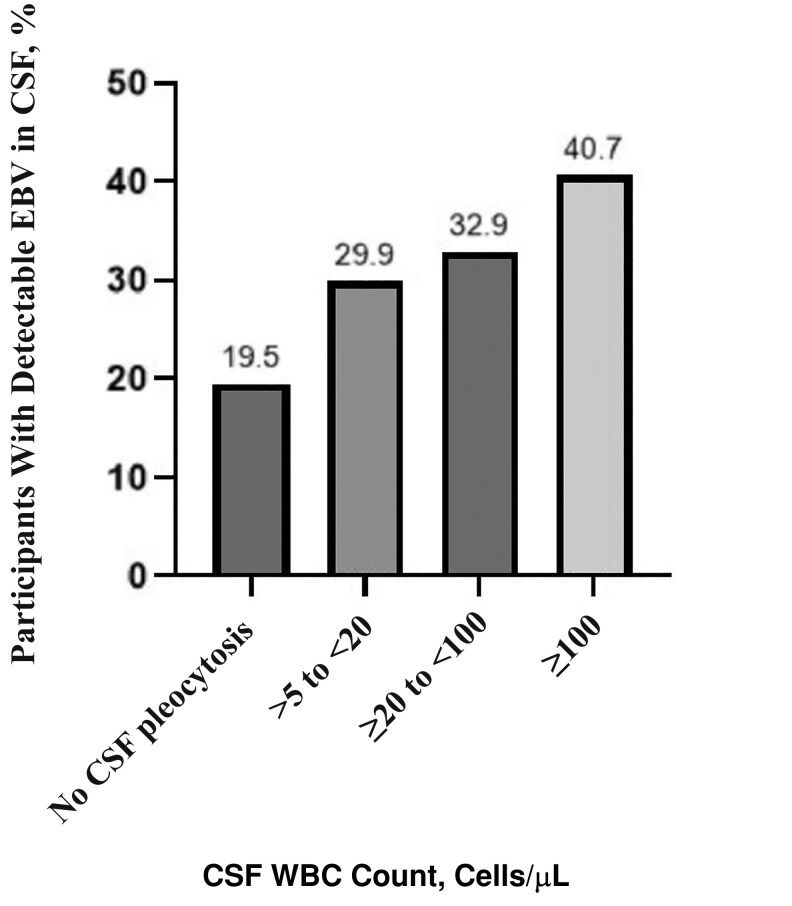
Proportion of study participants with evidence of Epstein-Barr virus (EBV) central nervous system coinfection stratified by baseline cerebrospinal fluid (CSF) white blood cell (WBC) count.

More than a quarter of all participants had evidence of EBV CNS infection (152 of 581 [26.2%]), of whom 78 of 152 (51.3%) had evidence of EBV CNS monoinfection, and 74 of 152 (48.7%) had evidence of EBV CNS coinfection with a second confirmed pathogen: EBV–cryptococcal meningitis coinfection (n = 27), EBV–bacterial meningitis coinfection (n = 17), EBV–definite tuberculous meningitis coinfection (n = 18), or EBV detected with another viral pathogen (n = 12). The median EBV CSF VL (IQR) was 1885 (575–14 750) copies/mL. EBV CNS coinfection was strongly associated with being an adult (present in 28.7% of adults vs 7.5% of children), HIV seropositivity (present in 30.5% of participants living with HIV vs 14.3% of HIV-negative participants), and CSF pleocytosis (present in 33.2% of participants with vs 19.5% of those without CSF pleocytosis) (all *P* < .001) (Table 1 and Figure 1).

### Survival Analyses

The overall in-hospital mortality rate was 30.3% (172 of 568 participants) and varied significantly across the meningitis diagnostic subgroups (*P* = .009); it was 44% with tuberculous meningitis (33 of 75), 41% with bacterial meningitis (19 of 46), 32% with cryptococcal meningitis (22 of 69), 28% with viral meningitis (40 of 141), and 25% with other suspected meningitis (58 of 237).

The in-hospital mortality rate was similar among participants living with HIV (32% [122 of 385]) and those living without HIV (27% [32 of 118)]) (*P* = .35). Among 385 participants living with HIV, the mortality rates were comparable between participants with EBV CNS coinfection (35% [40 of 116]) and those without EBV CNS coinfection (31% [82 of 269]) (*P* = .44). Among 118 participants living without HIV, the mortality rate was higher in those with EBV CNS coinfection (47% [8 of 17]) than in those without EBV CNS coinfection (24% [24 of 101]) (*P* = .05). Overall 38.3% (57 of 149) of participants with EBV CNS coinfection died in the hospital, compared with 27.4% (115 of 419) of those without EBV CNS coinfection (*P* = .01). On univariable analysis, EBV CNS infection was associated with the in-hospital mortality rate (crude odds ratio [OR], 1.64 [95% confidence interval (CI), 1.10–2.43]; *P* = .014). Following adjustment for sex, age, and HIV status, there was no longer evidence of an association (adjusted OR [aOR], 1.31[.85–2.03]; *P* = .22) (Table 3).

**Table 3.** Multivariable Analysis of the Association Between Epstein-Barr Virus Central Nervous System Infection and In-Hospital Mortality Rate Using Logistic Regression Modeling^a^

	In-Hospital Mortality Rate, No. of Deaths/Total No. (%)	cOR for In-Hospital Mortality Rate (95% CI)	*P* Value	aOR for In-Hospital Mortality Rate^b^ (95% CI)	*P* Value
**Total cohort**	172/568 (30.3)	…	…	…	…
EBV CNS infection present					
No	115/419 (27.4)	1 (Reference)	…	1 (Reference)	…
Yes	57/149 (38.3)	1.64 (1.10–2.43)	.01	1.31 (.85–2.03)	.22
CNS EBV VL					
<1885 copies/mL	23/76 (30.3)	1.14 (.67–1.96)	.62	1.04 (.59–1.84)	.90
≥1885 copies/mL	34/73 (46.6)	2.30 (1.39–3.83)	.001	1.70 (.95–3.02)	.07

Abbreviations: aOR, adjusted odds ratio; CI, confidence interval; CNS, central nervous system; cOR, crude odds ratio; EBV, Epstein-Barr virus; VL, viral load.

^a^Goodness-of-fit was assessed using the Hosmer-Lemeshow test (*P* > .05).

^b^Adjusted for age, sex and human immunodeficiency virus status. Cerebrospinal fluid inflammatory markers (white blood cell count and glucose and protein levels) and diagnostic group were not adjusted for as potentially on the causal pathway.

When EBV VL was analyzed as a continuous variable, we found that for every increase of 10 000 copies/mL in the CSF EBV burden, the odds of in-hospital mortality increased by 14% (crude OR, 1.14 [95% CI, 1.02–1.28]; *P* = .03); however, there was no evidence of an association following adjustment for age, sex, and HIV status (aOR, 1.07 [.95–1.20]; *P* = .29).

**Figure 2.**
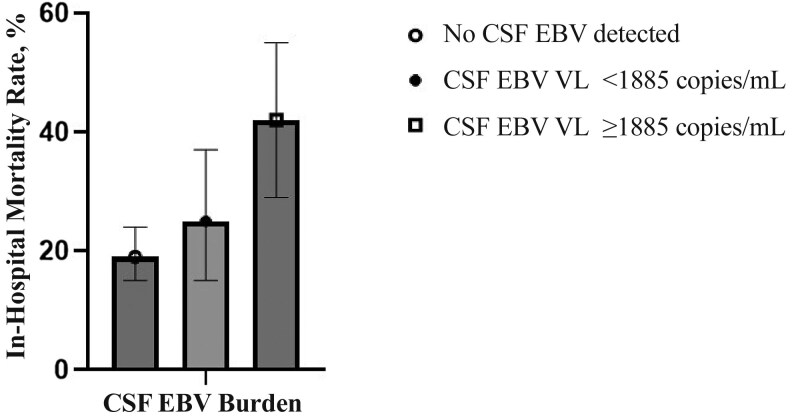
In-hospital mortality rate stratified by cerebrospinal fluid (CSF) Epstein-Barr virus (EBV) burden among 568 adults and children with suspected meningitis. Abbreviation: VL, viral load.

Participants with a higher burden of EBV CNS coinfection had a higher mortality rate: 46.6% of participants (34 of 73) with a CSF EBV VL ≥1885 copies/mL died during hospitalization, compared with 30.3% (23 of 76) with a CSF EBV VL <1885 copies/mL, and 27.4% (115 of 419) with no evidence of EBV infection (*P* = .005). At univariable analysis, participants with a high EBV burden (≥1885 copies/mL) had >2 times the odds of in-hospital mortality compared with participants with no evidence of EBV CNS coinfection (cHR, 2.30 [95% CI, 1.39–3.83]); however, there was no evidence of association following adjustment for sex, age, and HIV status (Table 3 and Figure 2).

### Subgroup Analyses

Our exploratory subgroup analyses found some evidence that the association between EBV CNS infection and in-hospital mortality rate varied according to meningitis diagnostic group. Following adjustment for age, sex, and HIV status, among participants with cryptococcal meningitis, the point estimate indicated that EBV coinfection was associated with reduced in-hospital mortality rate (aOR, 0.56 [95% CI, .15–2.04]); conversely, among participants with tuberculous meningitis (aOR, 2.06 [.74–5.75]) and bacterial meningitis (1.27 [.28–5.69]), the point estimates indicated that EBV coinfection was associated with increased in-hospital mortality rate. However, there was no statistical evidence for these associations (Table 4), using formal likelihood ratio test testing for interaction (*P* = .76).

**Table 4.** Subgroup Analysis of the Association Between Epstein-Barr Virus Central Nervous Infection and In-Hospital Mortality Rate by Diagnostic Group, Using Logistic Regression Modeling

**Suspected Meningitis Group**	**In-Hospital Mortality Rate,** **No. of Deaths/Total No. (%)**	**cOR for In-Hospital Mortality Rate** **(95% CI)**	***P* Value**	**aOR for In-Hospital Mortality Rate** ^a^ **(95% CI)**	***P* Value**
Cryptococcal meningitis without EBV	13/42 (30.9)	1 (Reference)	…	1 (Reference	…
Cryptococcal meningitis with EBV coinfection	9/27 (33.3)	1.11 (.40–3.13)	.84	0.56 (.15–2.04)	.38
Bacterial meningitis without EBV	11/28 (39.3)	1 (Reference)	…	1 (Reference)	…
Bacterial meningitis with EBV coinfection	8/18 (44.4)	1.24 (.37–4.10)	.73	1.27 (.28–5.69)	.75
Tuberculous meningitis without EBV	18/49 (36.7)	1 (Reference)	…	1 (Reference)	…
Tuberculous meningitis with EBV coinfection	15/26 (57.7)	2.35 (.89–6.20)	.08	2.06 (.74–5.75)	.16
Viral meningitis without EBV	28/102 (27.4)	1 (Reference)	…	1 (Reference)	…
Viral meningitis with EBV coinfection	12/39 (30.8)	1.17 (.52–2.63)	.70	1.00 (.40–2.49)	1.00
Other suspected meningitis without EBV	45/198 (22.7)	1		1	
Other suspected meningitis with EBV coinfection	12/39 (33.3)	1.70 (.81–3.58)	.16	1.35 (.57–3.18)	.49

Abbreviations: aOR, adjusted odds ratio; CI, confidence interval; cOR, crude odds ratio; EBV, Epstein-Barr virus.

^a^Adjusted for age, sex and human immunodeficiency virus status. Cerebrospinal fluid inflammatory markers (white blood cell count and glucose and protein levels) and diagnostic group were not adjusted for as potentially on the causal pathway.

## DISCUSSION

In our cohort of 581 adults and children presenting with suspected meningitis, EBV CNS infection was common, occurring in more than a quarter of participants. EBV occurred both as a monoinfection and with a range of other CNS pathogens, and it was strongly and positively associated with CSF inflammation and with being HIV positive. Following multivariable analyses, EBV CNS infection overall was not associated with the in-hospital mortality rate. There was weak evidence that having a high burden of EBV CNS coinfection was associated with increased odds of in-hospital mortality, despite adjustment for age, sex, and HIV status. There was some evidence that the association between EBV CNS coinfection status and mortality rate varied according to meningitis diagnostic group.

Our prevalence estimate of EBV CNS infection among patients presenting with suspected meningitis is comparable to findings in previously published studies set in Africa [10, 13–17]. Similarly, our data suggesting that high-burden EBV CNS coinfection is associated with increased mortality rate are in agreement with Malawian data from adults diagnosed with bacterial meningitis, in whom high EBV VL CSF detection was associated with an increased in-hospital mortality rate [13].

Our finding that the prognostic significance of EBV coinfection may vary by meningitis diagnostic subgroup is novel. In this exploratory analysis, among participants with cryptococcal meningitis, the point estimate indicated that EBV coinfection was associated with a 44% reduced odds of in-hospital mortality, while in participants with tuberculous meningitis the point estimate indicated that EBV coinfection was associated with more than double that odds. Our finding of a differential association between EBV CNS coinfection and survival between participants with cryptococcal meningitis versus other forms of meningitis has not previously been reported. While this finding is novel, it is not unexpected. We know that among patients being treated for HIV-associated cryptococcal meningitis, CSF pleocytosis—reflective of a heightened host-mediated immune response—is protective [26]. Conversely, tuberculous meningitis and bacterial meningitis are highly inflammatory meningitis syndromes, and there is good evidence that adjunctive steroids, to effectively dampen the host immune response, are associated with improved outcomes [27].

In light of our data, therefore, we hypothesize that among patients presenting with suspected meningitis, PCR detection of EBV in the CSF is most likely indicative of CSF inflammation and effective trafficking of lymphocytes—containing EBV—across the blood brain barrier and into the CSF during meningitis. Rather than acting as a true symbiotic or pathogenic virus, EBV CNS coinfection in the context of meningitis, should therefore be regarded as a “bystander” in the CSF, reflective of a heightened host-mediated immune response.

The potential additive proinflammatory role of EBV interleukin 10 (IL-10) in the context of coinfection should also be considered. EBV IL-10, a lytic phase protein, is a homologue of human IL-10. Human IL-10 is an immunomodulatory anti-inflammatory cytokine, and its role is to suppress and control the magnitude of the host response in order to avoid excessive immune activation and its consequences [28]. In active EBV infection, EBV IL-10 is produced. As a functional homologue of human IL-10, EBV IL-10 competes with human IL-10–binding sites, and acting as an antagonist it leads to a proinflammatory host response. This association has previously been reported to be of importance in a range of autoimmune conditions [29, 30], but one could also hypothesize that high-level EBV CNS coinfection could potentiate inflammation and therefore be associated with poor outcomes in meningitis phenotypes associated with excessive inflammation, including tuberculous meningitis.

Our study has several strengths. This is the largest study to date to describe the prevalence of EBV CNS infection among patients presenting with suspected meningitis. Our prevalence estimate of 26% is consistent with findings of other studies, but given that our sample size (n = 581) is 4 times greater than that of any previous study in Africa, the precision of our estimate is better. Furthermore, inclusion of both adults and children from 2 countries improves the generalizability of our estimates.

Our study also has several limitations. First, 67% of our cohort presenting with meningitis did not have a microbiologically confirmed diagnosis. This proportion is consistent with other meningitis cohorts from comparable resource-limited settings [2, 3] and reflects the paucity of sensitive meningitis diagnostics available; however, we do recognize the risk of misclassifying nonmeningitis cases as meningitis. The fact that not all participants received all diagnostic tests, usually due to lack of sample availability, further compounded this limitation within our cohort. Due to this information bias, therefore, our EBV prevalence estimate likely represents an underestimate of the actual EBV burden among patients with confirmed meningitis. Second, for our subgroup analyses, we formulated 5 composite diagnostic groups, which would have reduced the precision and specificity of our subgroup analyses. Our relatively small sample size was a major limitation in our exploratory subgroup analyses. While there was a signal that the association between EBV CNS infection and in-hospital mortality rate varied according to meningitis diagnostic group, the lack of outcome events in each diagnostic group precluded more robust dose-response analyses. The fact that 13 study participants were missing the survival status outcome further impeded these analyses. Validation of our findings within a larger cohort is therefore required.

In summary, we report that PCR detection of EBV in the CSF is common among children and adults presenting with meningitis. It was more common among participants living with HIV, and there was a very strong association between EBV CNS coinfection and CSF inflammation (CSF WBC count and protein level). Taken together with the contrasting associations with mortality rate, our data suggest that EBV is a bystander virus that reflects heightened CSF inflammation among patients with meningitis, rather than a clinically significant infection. Larger longitudinal studies are required to understand the association between EBV, CSF inflammation, and outcome and to investigate how mechanistic pathways may differ across meningitis subgroups.

## Supplementary Material

ofaf660_Supplementary_Data

## Supplementary Data

Supplementary materials are available at *Open Forum Infectious Diseases* online. Consisting of data provided by the authors to benefit the reader, the posted materials are not copyedited and are the sole responsibility of the authors, so questions or comments should be addressed to the corresponding author.
