# Supplementary figures and images for: Epstein-Barr Virus Central Nervous System Infections and Mortality Risk in Patients Presenting With Suspected Meningitis: Results From the Botswana National Meningitis Survey and the Harare Meningitis Aetiology Study
Source: Open Forum Infect Dis. 2025 Oct 23;12(12):ofaf660. doi: 10.1093/ofid/ofaf660 (PMC12661572; doi:10.1093/ofid/ofaf660)

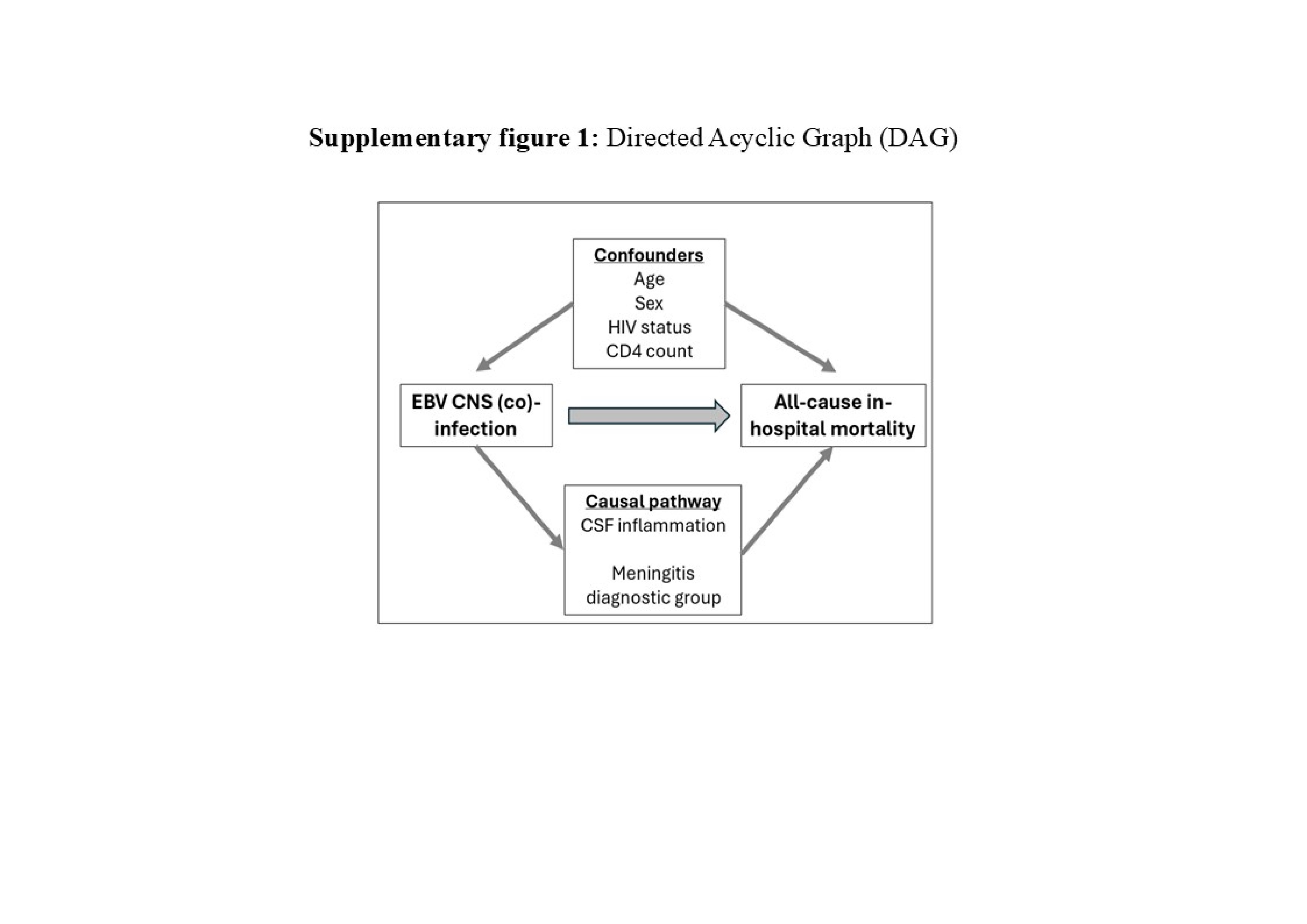

Supplement: ofaf660_Supplementary_Data [file ofaf660_supplementary_data.jpeg]
